# Draft Genome Sequence of a New *Oscillospiraceae* Bacterium Isolated from Anaerobic Digestion of Biomass

**DOI:** 10.1128/MRA.00507-20

**Published:** 2020-07-02

**Authors:** Javier Pascual, Sarah Hahnke, Christian Abendroth, Thomas Langer, Patrice Ramm, Michael Klocke, Olaf Luschnig, Manuel Porcar

**Affiliations:** a Darwin Bioprospecting Excellence, S.L., Paterna, Valencia, Spain; b Leibniz Institute for Agricultural Engineering and Bioeconomy e.V. (ATB), Department Bioengineering, Potsdam, Germany; c University of Oldenburg, Department of Human Medicine, Oldenburg, Germany; d Robert Boyle Institut e.V., Jena, Germany; e Institute of Waste Management and Circular Economy, Technische Universität Dresden, Pirna, Germany; f Institute of Agricultural and Urban Ecological Projects affiliated to Berlin Humboldt University (IASP), Berlin, Germany; g Bio H2 Umwelt GmbH, Jena, Germany; h Institute for Integrative Systems Biology (I2SysBio), University of Valencia-CSIC, Paterna, Valencia, Spain; University of Southern California

## Abstract

Here, we present the genome sequence and annotation of the novel bacterial strain HV4-5-C5C, which may represent a new genus within the family *Oscillospiraceae* (order *Eubacteriales*). This strain is a potential keystone species in the hydrolysis of complex polymers during anaerobic digestion of biomass.

## ANNOUNCEMENT

In the past few years, efforts have been undertaken to characterize new species isolated from anaerobic digesters (1, 2). However, few articles have focused on microorganisms isolated from acidic pretreatment stages. We present here the draft genome sequence of the bacterial strain HV4-5-C5C, which was isolated from the acidification stage of a mesophilic two-stage laboratory-scale leach bed system using as the sole substrate freshly cut grass taken from a meadow in Jena, Germany (50°51′55.4″N, 11°35′56.1″E). Isolation of the strain was performed after the diluted hydrolysate was reincubated with microcrystalline cellulose as the sole carbon source. After incubation, the hydrolysate was diluted 10^5^-fold, plated on BBL Columbia agar (BD Biosciences) supplemented with 5% laked horse blood, and cultivated under anoxic conditions at 37°C. For purification, single colonies were picked and restreaked several times.

After cultivation in brain heart infusion broth (Carl Roth) supplemented with yeast extract, DNA was extracted and purified using the Gentra Puregene Yeast/Bact. kit (Qiagen) and the NucleoSpin genomic DNA (gDNA) cleanup kit (Macherey-Nagel). We constructed a Nextera XT library from the total genomic DNA and sequenced it using the Illumina NextSeq 500 platform (150-bp paired-end reads). The raw reads were filtered (quality [Q], >20; minimum length, >50 nucleotides [nt]) with BBTools v37.10, yielding 23.48 million paired-end sequences with a mean Q value of 32.93. Genome assembly was conducted with the software SPAdes v3.13.0 (3). A total of 72 contigs were obtained (length, ≥300 nt), covering a total genome size of 2,867,854 nt with an estimated GC content of 53.25%. The largest contig was 296,629 nt, and the *N*_50_ value was 134,989 nt. The final coverage of the genome was 2,457×.

The assembled sequences were annotated using the NCBI Prokaryotic Genome Annotation Pipeline (4) and the RAST toolkit (5) implemented in PATRIC (6). The genome of strain HV4-5-C5C harbors 2,462 genes, including 2,364 coding DNA sequences (CDSs), 45 pseudogenes, 3 rRNAs organized in a single operon, 47 tRNAs, and 3 noncoding RNAs (ncRNAs).

The genome harbors 88 different glycoside hydrolases and 2 polysaccharide lyases (7). Strain HV4-5-C5C might be able to carry out alcoholic fermentation as well as the synthesis of lactate, formate, and acetate (Table 1). Therefore, strain HV4-5-C5C may be a keystone species in the hydrolysis of complex polymers as well as in the acidogenesis and acetogenesis steps.

**TABLE 1:** Metabolic pathways involved in the acidogenesis and acetogenesis steps

Metabolic pathway	Enzyme(s) (EC no.)
Alcoholic fermentation	Ethanol dehydrogenase (1.1.1.1)
Formation of lactate	l- and d-lactate dehydrogenases (1.1.1.27; 1.1.1.28)
Conversion of pyruvate to acetyl-CoA^*a*^ and formate	Pyruvate formate lyase (2.3.1.54)
Conversion of acetyl-CoA to acetate	Acetate kinase (2.7.2.1) and phosphate acetyltransferase (2.3.1.8)

a CoA, coenzyme A.

The type strain most closely related to HV4-5-C5C was Mageeibacillus indolicus CCUG 59143, sharing 90.31% 16S rRNA gene sequence similarity (EzBioCloud, v2020.02.25 [8]). Calculation of the average nucleotide identity (JSpecies tool v3.4.7) resulted in a value of 65.10% between both strains (9, 10).

The average amino acid identity (11) and the percentage of conserved proteins (12) calculated for strain HV4-5-C5C and *M. indolicus* CCUG 59143, the type species of the genus *Mageeibacillus*, were 15.16% and 25.13%, respectively. Hence, we can assume that strain HV4-5-C5C may represent a new genus within the family *Oscillospiraceae* (order *Eubacteriales*) (11, 12). Default parameters were used for all software unless otherwise specified.

### Data availability.

Strain HV4-5-C5C was deposited at the German Collection of Microorganisms and Cell Cultures under the designation DSM 103941. This whole-genome sequencing (WGS) project has been deposited at DDBJ/ENA/GenBank under the accession number JAAVLZ000000000.1. The raw sequence reads are deposited under SRA accession number SRR11413021. The WGS and SRA records are associated with BioProject accession number PRJNA614915.
